# 

**Published:** 1886-01

**Authors:** 


CONTENTS.

Vol. 33 No. I

| | PAGE. | | PAGE. |
|---|---|---|---|
| Announcement | 1 | Indigestible Food | 28 |
| Our Future | 1 | Gelatine | 28 |
| The Prayer Cure | 2 | Frying | 28 |
| Pneumonia | 7 | Roast Meats | 29 |
| Malarial Fever | 9 | To Fry Potatoes | 29 |
| Diphtheria | 13 | Rice Pudding | 29 |
| Drinking Water | 16 | An Economical Pudding | 29 |
| The Use of Ivy | 18 | Test of Butter | 29 |
| Cocoaine | 19 | Scarlet Fever | 31 |
| Compulsory Vaccination | 21 | Danger From Fig Tree Twigs | 31 |
| Danger of Food and Drink | 23 | The Hog | 31 |
| The Hydraphobiac Scare | 24 | Pat. Cooney Hurt by Prayer | 31 |
| Reform of the Lunacy Laws | 26 | Frost Bitten Toes | 31 |
| Fresh Water | 28 | | |

